# Community-Based Double Reflex Screening Reveals Widespread Hepatitis D Viremia Among Hepatitis B Surface Antigen Carriers in Pakistan

**DOI:** 10.7759/cureus.85200

**Published:** 2025-06-01

**Authors:** Minaam Abbas, Zaigham Abbas, Muhammed Y Sharif

**Affiliations:** 1 Internal Medicine, Addenbrooke's Hospital, Cambridge University Hospitals NHS Foundation Trust, Cambridge, GBR; 2 Gastroenterology and Hepatology, Dr. Ziauddin University Hospital, Karachi, PAK; 3 Hepatology, Ferozsons Laboratories Limited, Karachi, PAK

**Keywords:** anti-hdv antibody, hdv rna, hepatitis b, hepatitis d, reflex testing

## Abstract

Background

Hepatitis D, caused by the hepatitis delta virus (HDV), poses a significant public health concern in many parts of the world. However, HDV screening remains suboptimal in most regions, leading to underdiagnosis of the infection. This study aimed to determine the prevalence of HDV infection through double reflex testing of hepatitis B surface antigen (HBsAg)-positive individuals at screening camps in two high-prevalence towns in the eastern part of Balochistan province, Pakistan.

Methods

A total of 1,643 individuals were screened for HBsAg and antibodies to the hepatitis C virus (anti-HCV). Those who tested positive for HBsAg were further tested for antibodies to HDV (anti-HDV). Samples positive for anti-HDV were subsequently analyzed for HDV RNA using real-time PCR.

Results

Of the 1,643 individuals screened, 277 (16.9%) tested positive for HBsAg and 227 (13.8%) for anti-HCV. Among the HBsAg-positive individuals, 186 (67.1%) were positive for anti-HDV antibodies, including 133 men and 53 women, with a mean age of 32.2 ± 11.3 years. HDV RNA was detectable in 108 (58.1%) of the anti-HDV-positive individuals, with mean HDV RNA levels of log₁₀ 6.69 ± 1.35 IU/mL. Of the 277 HBsAg-positive individuals, five also tested positive for anti-HCV; one of these was positive for anti-HDV but negative for HDV RNA.

Conclusions

Reflex testing of HBsAg-positive individuals in this HDV-endemic area showed that 67% had been exposed to HDV, and 58.1% of those with anti-HDV antibodies had active viremia. These findings underscore the importance of implementing double reflex testing - screening HBsAg-positive individuals for anti-HDV antibodies, followed by HDV RNA testing in those who are anti-HDV positive - in regions with high HDV prevalence.

## Introduction

Chronic hepatitis delta virus (HDV) infection is found in 4.5-14.5% of hepatitis B surface antigen (HBsAg)-positive individuals and affects an estimated 12-60 million people globally, according to three recently published meta-analyses [1-3]. However, the true global prevalence of HDV remains unclear, even in these meta-analyses, due to insufficient data. Hepatitis D is recognized as the most severe form of viral hepatitis [4]. HDV is a satellite virus of hepatitis B virus (HBV) and infects individuals either through coinfection or superinfection. It significantly accelerates liver disease progression, leading to advanced fibrosis, cirrhosis, and complications such as end-stage liver disease and hepatocellular carcinoma [5]. According to WHO, at least 5% of individuals chronically infected with HBV are also infected with HDV [6].

Chronic viral hepatitis poses a major public health challenge in Pakistan. An estimated 13 million people in the country are living with hepatitis B or C, making these among the most widespread infections nationwide [7]. The high prevalence has been attributed to factors such as a fragmented healthcare infrastructure, limited access to medical services, poor hygiene practices, and high-risk behaviors, including needle sharing and unprotected sex. These challenges, combined with low hepatitis B vaccination coverage, inadequate screening of pregnant women for HBV, poor public awareness, and limited access to treatment and care, have contributed to a substantial burden of HDV infection. Furthermore, the lack of awareness surrounding HDV has led to the underestimation of its true impact among individuals infected with HBV.

Pakistan is among the countries with the highest burden of hepatitis D, with approximately 16-18% of HBsAg-positive individuals testing positive for anti-HDV antibodies [8]. HDV is particularly prevalent in the eastern districts of Balochistan and neighboring regions of Sindh and Punjab provinces. Despite this, HDV screening efforts remain inadequate. This study aimed to estimate the prevalence of HDV infection by employing a double reflex testing approach among HBsAg-positive individuals who attended community screening camps in Usta Muhammad and Dera Allah Yar, two adjacent high-prevalence towns in the eastern part of Balochistan province.

## Materials and methods

Study design and setting

**Figure 1:**
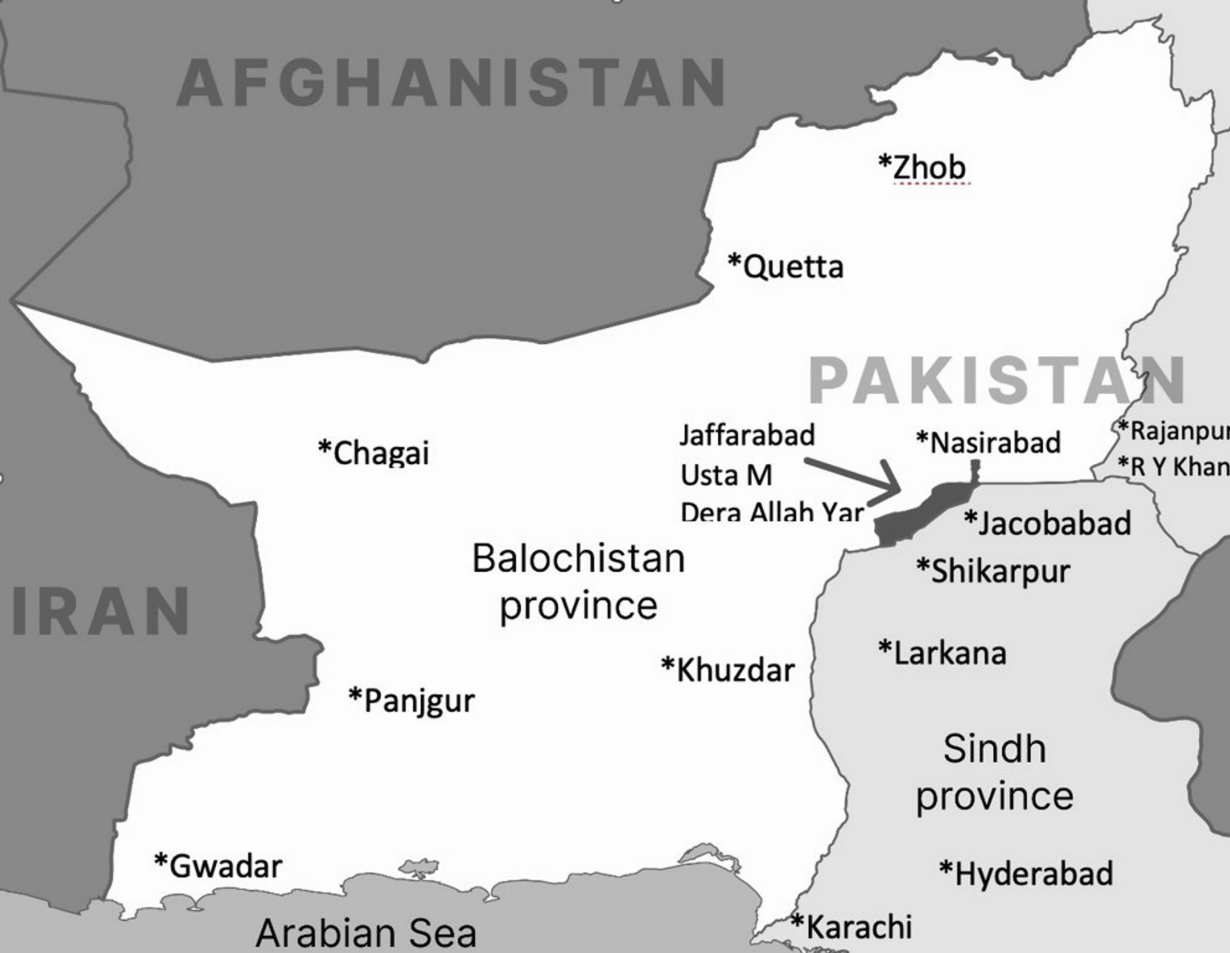
Study location in Pakistan Double reflex testing was conducted among HBsAg-positive individuals who attended screening camps in Usta Muhammad and Dera Allah Yar, towns in eastern Balochistan Province. HBsAg, hepatitis B surface antigen Figure created by Minaam Abbas using Adobe Illustrator

A hepatitis awareness campaign was conducted in November and December 2021 in Usta Muhammad and Dera Allah Yar - two neighboring towns in eastern Balochistan, Pakistan (Figure 1). These towns have a combined population of approximately 300,000. As part of the community-based awareness and screening initiative, participants received education on hepatitis transmission, and free testing for hepatitis B and C was offered at local primary healthcare centers. The campaign involved experienced community health workers, physicians, and paramedical staff.

This cross-sectional study was designed to assess the prevalence of HDV infection among HBsAg-positive individuals using a double reflex testing strategy: initial anti-HDV antibody screening followed by HDV RNA PCR testing for those who tested positive for anti-HDV antibodies.

Objectives

The primary objective of this study was to estimate the prevalence of HDV viremia using a double reflex testing strategy in individuals who tested positive for HBsAg. A secondary objective was to investigate any correlation between HDV RNA viral load and patient age or alanine aminotransferase (ALT) levels.

Participants

Eligible participants included adults and adolescents aged 12 years and older who had no prior history of hepatitis B or C testing and who attended the community screening camps. Informed consent was obtained from all participants or, in the case of minors, from their accompanying parents or guardians. Individuals were excluded if they had a known history of hepatitis B or C treatment or if demographic or clinical data were incomplete, such as missing or inaccurate age information or incomplete information regarding past hepatitis infection.

Sample size

A convenience sample of 1,643 participants was enrolled based on attendance at the screening camps. This sample size was deemed sufficient to estimate the prevalence of HDV with a 95% confidence interval and a 5% margin of error, based on previously reported regional HBsAg and anti-HDV positivity rates [8].

Laboratory methods

Screening for HBsAg and anti-HCV antibodies was conducted using WHO-prequalified Bioline™ point-of-care immunochromatographic rapid tests (Abbott Diagnostics Korea Inc., Gyeonggi-do, Republic of Korea). Capillary blood was collected via finger prick, and tests were conducted according to the manufacturer’s instructions. Results were interpreted at 15 minutes; the presence of two bands indicated a positive result. These assays report 99.3% sensitivity and 100% specificity for anti-HCV detection and 100% sensitivity and specificity for HBsAg detection [9,10].

HBsAg-positive individuals underwent further testing for anti-HDV antibodies using a commercial enzyme immunoassay (Diasorin, Italy), which has 100% sensitivity and specificity for anti-HDV detection [11]. Briefly, serum samples were centrifuged at 3,000 rpm for 10 minutes and stored at −80°C until analysis. A cutoff optical density value of ≥1.0 was used to define anti-HDV positivity.

HDV RNA detection

HDV RNA was detected via real-time PCR using the Rotor-Gene Q Real-Time PCR system (QIAGEN GmbH, Hilden, Germany). RNA extraction was carried out using the QIAamp Viral RNA Mini Kit (QIAGEN), and amplification was performed with HDV-specific primers targeting the ribozyme region. This assay offers excellent sensitivity (<100 IU/mL) and 100% specificity [12]. PCR cycling conditions were as follows: 50°C for 30 minutes, 95°C for 15 minutes, followed by 45 cycles of 95°C for 15 seconds and 60°C for 60 seconds. HDV RNA levels were quantified and expressed as log₁₀ IU/mL using a standard curve.

Liver biochemistry (subgroup analysis)

Liver biochemistry tests were conducted on a subgroup of 64 HDV RNA-positive individuals who provided additional consent. Parameters measured included ALT, bilirubin, gamma-glutamyl transferase (GGT), and alkaline phosphatase (ALP), using the Architect c8000 analyzer (Abbott Laboratories, Chicago, Illinois, USA).

Statistical analysis

Data were analyzed using IBM SPSS Statistics for Windows, Version 26.0 (Released 2019; IBM Corp., Armonk, NY, USA). Descriptive statistics were expressed as mean ± SD (for variables such as age and viral load), median (range), or frequencies and percentages. The Mann-Whitney U test was used for non-normally distributed continuous variables, and the chi-square or Fisher’s exact test was used for categorical variables. Pearson’s correlation test was applied to assess correlations between HDV RNA viral load and age or ALT levels. A two-tailed p-value <0.05 was considered statistically significant.

Quality assurance

All staff involved in the study received standardized training in sample collection and testing protocols. Each PCR run included both negative and positive controls. Laboratory technicians were blinded to clinical data, and 10% of samples were retested to ensure reproducibility and quality control.

Ethical considerations

Written informed consent was obtained from all participants. Individuals who tested positive were referred for free clinical follow-up and care. The study protocol was approved by the Ethics Research Committee of Ziauddin University (approval number 7821023ZAGE) and conducted in accordance with the Declaration of Helsinki, Good Clinical Practice guidelines, and local regulatory requirements.

## Results

**Figure 2:**
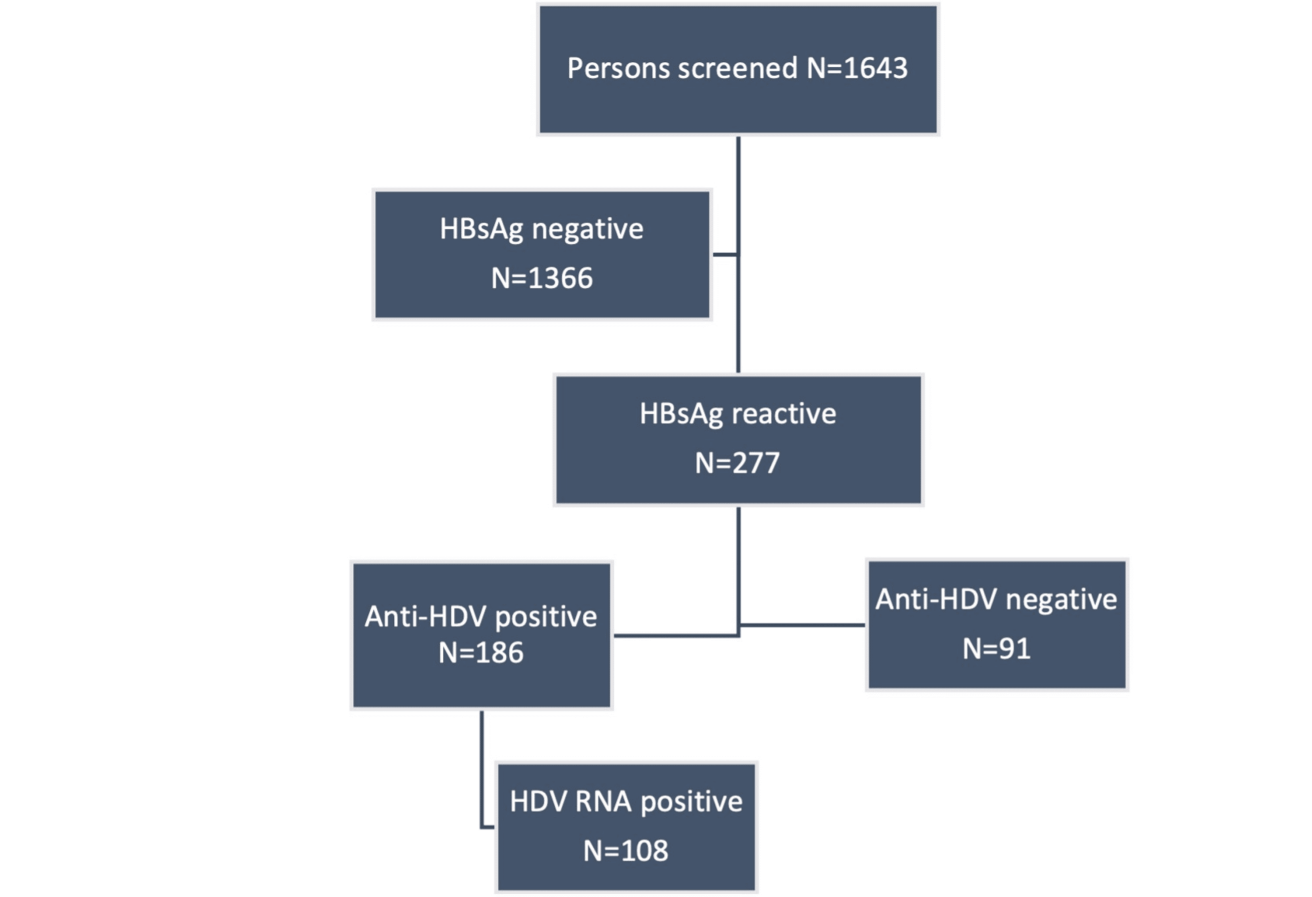
Study disposition Of the 1,643 individuals screened, 277 were HBsAg positive. Among these, 186 tested positive for anti-HDV antibodies, and of those, 108 were confirmed to have HDV RNA viremia. HBsAg, hepatitis B surface antigen; HDV, hepatitis delta virus

A total of 1,959 individuals attended the community screening camps. Of these, 316 were excluded due to prior knowledge of their serological status, a history of hepatitis treatment, or incomplete demographic data (e.g., inability to recall exact age or type of previous hepatitis infection). Consequently, 1,643 individuals were screened. Among these, 277 individuals (16.8%) tested positive for HBsAg, with 181 of them being male (65.3% of HBsAg-positive individuals) (Figure 2).

The mean age of HBsAg-positive participants was 31.6 ± 12.0 years, with a median age of 30 years (range: 12-66 years). Anti-HDV antibodies were detected in 186 individuals (133 males), representing 67.1% of those who tested positive for HBsAg and 11.3% of the total screened population. Among the anti-HDV-positive individuals, HDV RNA was detectable in 108 cases (58.1%). Specifically, HDV RNA was found in 64 out of 114 (56.1%) HBsAg-positive individuals from Dera Allah Yar and in 44 out of 72 (61.1%) from Usta Muhammad - both towns located in Balochistan, Pakistan. The clinical and demographic characteristics of these HDV RNA-positive individuals are summarized in Table 1.

**Table 1:** Characteristics of HDV viremic patients (n = 108) ALP, alkaline phosphatase; ALT, alanine aminotransferase; GGT, gamma-glutamyl transferase; HDV, hepatitis delta virus

Parameter	Value
Mean age (years), mean ± SD	30.8 ± 10.2
Median age (years), range	30 (13-60)
Age ≤40 years, N (%)	90 (83.3%) (p < 0.001)
Gender, N (%)	
Male	81 (75%)
Female	27 (25%)
Male-to-female ratio	3:1 (p < 0.001)
HDV RNA levels (log₁₀ IU/mL)	
Mean ± SD	6.69 ± 1.34
Median (range)	6.98 (3.92-8.95)
Liver biochemistry (N = 64)	
Total bilirubin (mg/dL), mean ± SD	1.17 ± 0.40
ALT (IU/L), mean ± SD	100.7 ± 50.8
ALT (IU/L), median (range)	85 (32-282)
ALT < 40 IU/L, N (%)	2 (3.1%)
GGT (IU/L), mean ± SD	34.6 ± 38.2
ALP (IU/L), mean ± SD	200.2 ± 100.7

**Figure 3:**
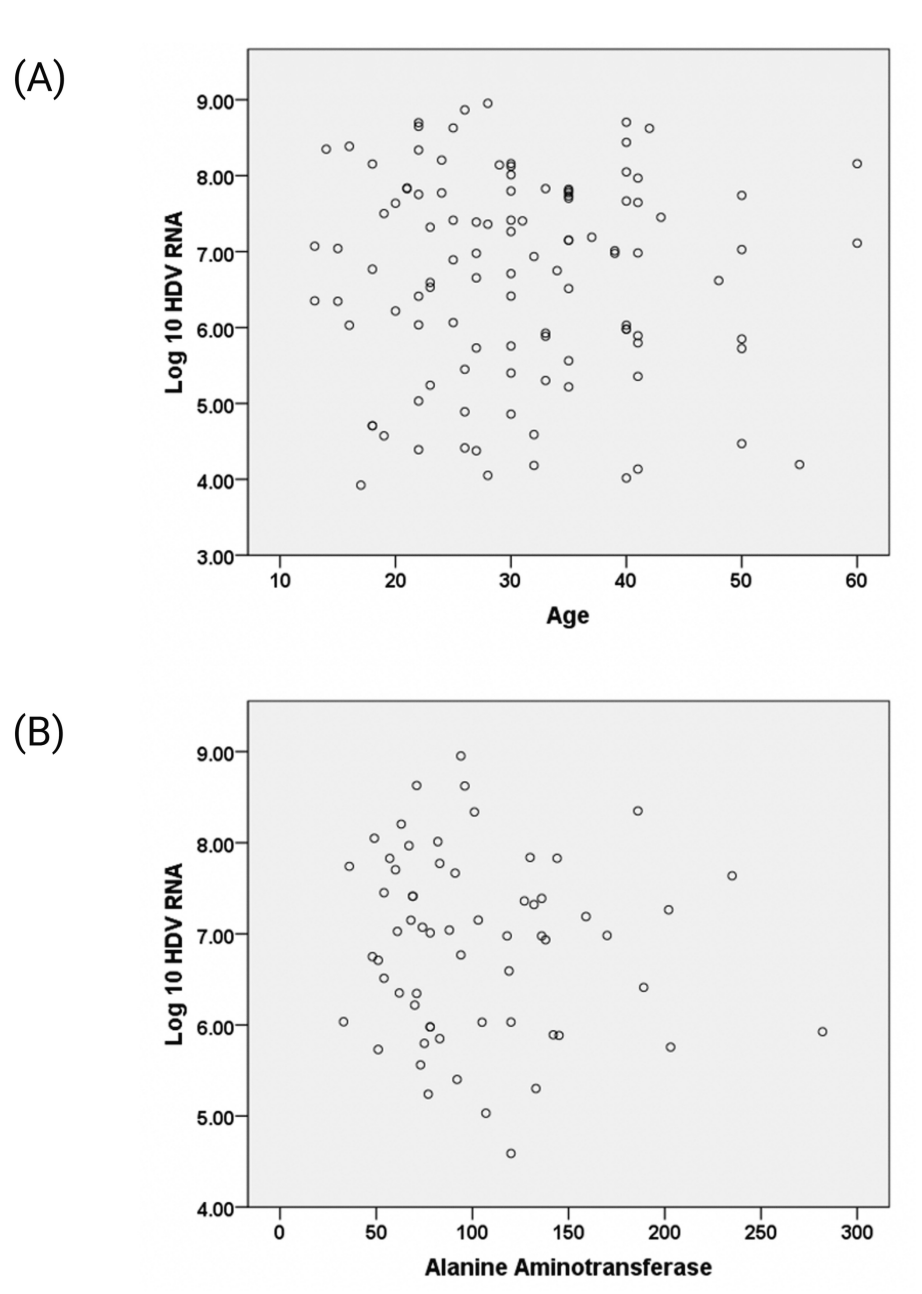
Distribution of HDV RNA levels by age (A) and ALT levels (B) There was no correlation between viral load and either age (p = 0.470) or ALT levels (p = 0.660). ALT, alanine aminotransferase; HDV, hepatitis delta virus

Among the HDV RNA-positive individuals, 81 (75%) were male. The mean HDV RNA level in this group was 6.69 ± 1.35 log₁₀ IU/mL, with a range of 3.92 to 8.95. The mean age of anti-HDV reactive individuals was 32.16 ± 11.32 years, compared to 30.54 ± 13.51 years in those who were anti-HDV negative. Among the anti-HDV-positive group, the mean age of patients with detectable HDV RNA was 30.8 ± 10.2 years, while it was 34.0 ± 12.3 years in non-viremic individuals (p = 0.121). The majority of viremic patients (90 out of 108; 83.3%) were aged 40 years or younger (Figure 3). However, no significant correlation was found between age and HDV RNA viral load (p = 0.470).

Liver biochemistry analysis was not part of the original study design; however, results became available for 64 HDV RNA-positive patients. The mean ± SD values were as follows: bilirubin, 1.17 ± 0.40 mg/dL; ALT, 100.7 ± 50.8 IU/L; GGT, 34.6 ± 38.2 IU/L; and ALP, 200.2 ± 100.7 IU/L. No correlation was observed between ALT levels and viral load (p = 0.660) (Figure 3). Notably, two patients with ALT levels below 40 IU/L had high viral loads, with log₁₀ HDV RNA levels of 6.04 and 7.74 IU/mL, respectively.

Anti-HCV antibodies were detected in 227 (13.8%) of the 1,643 individuals screened. Four individuals were co-infected with HBV (HBsAg-positive), and one patient was positive for anti-HCV, HBsAg, and anti-HDV but tested negative for HDV RNA.

## Discussion

Hepatitis D is a distinct and severe form of viral hepatitis that affects individuals already infected with HBV. Screening HBsAg-positive patients for hepatitis D is essential for early detection and management, which can help reduce complications and prevent further transmission. The presence of anti-HDV antibodies indicates prior exposure to HDV, while detectable HDV RNA confirms an active infection. The American Association for the Study of Liver Diseases recommends risk-based testing for HDV [13], whereas the European Association for the Study of the Liver advocates for universal testing in all HBsAg-positive individuals. Despite these guidelines, the adoption of testing remains suboptimal.

To improve the detection of undiagnosed HDV infections, experts have recently recommended a double-reflex testing approach. This involves automatically screening all HBsAg-positive individuals for anti-HDV antibodies, followed by HDV RNA testing for those who are anti-HDV positive [14]. This strategy is expected to yield more accurate estimates of HDV prevalence.

In Pakistan, 16-18% of HBsAg-positive individuals test positive for anti-HDV antibodies [8], with significantly higher rates reported in certain central districts, including the area where this study was conducted. However, the precise prevalence of HDV viremia in these regions has been unclear. Three earlier laboratory-based studies estimated detectable HDV RNA in 14.6% to 30% of cases [15-17]. These studies involved blood samples from Punjab, Sindh, and Khyber Pakhtunkhwa provinces, encompassing areas with both high and low prevalence. In contrast, our study focused on a specific region in Balochistan province, known for a high burden of hepatitis D, as many patients from this area sought care at our clinic.

Although a prior survey reported hepatitis B prevalence in Balochistan [18], data on HDV viremia in the region remain scarce. A study conducted several years ago on clinical laboratory data showed a 60% anti-HDV prevalence among HBsAg-positive blood samples from Larkana, a city in Sindh located near our study site [8]. In our community-based screening, 67% of HBsAg-positive individuals tested positive for anti-HDV antibodies, and 58% of these were actively infected, with detectable HDV RNA and elevated ALT levels. Many of these patients were potential candidates for treatment, identified through double point-of-care testing. Interestingly, we also found that a significant number of individuals had been exposed to HDV but were non-viremic, indicating recovery. HDV infection is a heterogeneous disease that can either progress or improve over time, with some patients eventually clearing the virus (becoming HDV RNA negative) [19].

The impact of hepatitis in Pakistan extends far beyond physical illness. It imposes a significant financial burden on individuals and families, driven by the high cost and limited effectiveness of treatment, as well as productivity losses from illness or death. Additionally, social stigma surrounding hepatitis can lead to discrimination and isolation of affected individuals due to widespread misconceptions about the disease.

Because viral hepatitis often presents without symptoms and screening typically occurs in hospital settings, many cases remain undiagnosed. Community-level educational outreach can be especially beneficial for raising awareness and encouraging at-risk groups to undergo testing. Such initiatives can help improve diagnosis, promote disease awareness, and facilitate timely management.

The alarming statistics from our study highlight the urgent need for increased public awareness and preventive efforts. More must be done. Adequate funding for educational campaigns, including vaccination programs and linkage-to-care initiatives for patients identified at screening camps, is essential. These efforts will help ensure equitable access to basic healthcare services across all regions and socioeconomic strata.

In our study, males were more frequently infected with hepatitis D than females - a trend consistent with previous findings from the region. This gender disparity may reflect the higher prevalence of hepatitis B among men, who are more often exposed to risk factors and may have greater susceptibility to chronic infection. Hormonal differences may also contribute to this pattern [20]. We found no significant correlation between viral load and either increasing age or ALT levels; notably, two patients with ALT levels below 40 IU/L still had high viral loads. This may be due to HDV’s ability to infect individuals across all ages and the variability in immune responses [21]. Previous studies in Pakistan have confirmed that HDV genotype 1 is the predominant strain [22]. Our study was limited to evaluating the double-reflex testing approach.

A key strength of this study is its community-based design, which enabled the identification of undiagnosed HDV cases using a robust double-reflex testing strategy to assess HDV viremia among anti-HDV-positive individuals. However, there were also limitations. Screening was not conducted door-to-door and was limited to a specific time frame, which may have excluded individuals living farther from the central screening location. Participation was voluntary and limited to those attending the awareness program, introducing the possibility of healthy volunteer bias. While our cohort may not fully represent the general population, the study’s main objective was to assess the prevalence of HDV viremia among anti-HDV-positive individuals and identify those requiring treatment. As a cross-sectional study, our findings offer a snapshot of the current situation but do not provide longitudinal insights.

## Conclusions

Our aim was to raise awareness of hepatitis D and promote community-based screening for viral hepatitis. During the educational program, we identified numerous HBsAg-positive individuals. Among these, 67.1% tested positive for anti-HDV antibodies, and 58.1% of the anti-HDV-positive group had detectable HDV RNA. Our community engagement was well-received and facilitated linkage to care for individuals who were previously unaware of their infection. Most patients were young, consistent with the early childhood acquisition of HDV in endemic areas. Our findings reinforce the urgent need for community education, screening, vaccination, and routine double-reflex testing for all HBsAg-positive individuals, particularly in HDV-endemic regions.
